# Sequential dual-organ living donation: selection, operative approach, and early donor outcomes at a Canadian center

**DOI:** 10.3389/ti.2026.16970

**Published:** 2026-09-07

**Authors:** Su Kah Goh, Nazia Selzner, Sunita K. Singh, Ian D. McGilvray, Chaya Shwaartz, Trevor W. Reichman, Nicolas Goldaracena, Jason Y. Lee, Markus Selzner, Gonzalo Sapisochin, Blayne Amir Sayed, Mark S. Cattral, Anand Ghanekar

**Affiliations:** 1 HPB and Transplant Unit, Department of Surgery, Austin Precinct, The University of Melbourne, Parkville, VIC, Australia; 2 Ajmera Transplant Centre, Toronto General Hospital, University Health Network, Toronto, ON, Canada; 3 Division of Gastroenterology, Department of Medicine, University Health Network and University of Toronto, Toronto, ON, Canada; 4 Division of Nephrology, Department of Medicine, University Health Network and University of Toronto, Toronto, ON, Canada; 5 Division of General Surgery, Department of Surgery, University Health Network and University of Toronto, Toronto, ON, Canada; 6 Division of General and Thoracic Surgery, The Hospital for Sick Children, Toronto, ON, Canada; 7 Division of Urology, Department of Surgery, University Health Network and University of Toronto, Toronto, ON, Canada; 8 Department of General and Abdominal Surgery, Hospital Universitario Vall d’Hebron, Barcelona, Spain

**Keywords:** donor safety and outcomes, living donor hepatectomy, living donor nephrectomy, living donor transplantation, Sequential dual organ donation

## Abstract

Sequential dual-organ living donation, defined as donation of a kidney and a partial liver graft by the same individual in separate operations, represents a rare extension of living donor transplantation. A donor-focused analysis was performed to characterize the donor population, the operative considerations posed by a second donor operation, and early donor outcomes at a single high-volume center. A retrospective review of all dual-organ living donors at our institute was performed. Twenty-four donors were identified (n = 10 liver-first and n = 14 kidney-first). The median interval between donations was 2 years (IQR 1.1–4.2). Twelve donors donated both organs anonymously, nine donated one organ anonymously and directed the other, and three directed both donations. Anonymous donation is a strong motivator in this unique population. Ten donors (42%) participated in kidney paired donation. Liver donations (left lateral segment (42%), left lobe (25%), and right lobe (33%)) were performed by an open approach. Laparoscopic nephrectomy was performed in all cases except two prior liver donors. Liver-after-kidney donors (n = 14) did not develop renal dysfunction after donation. Prior organ donation does not usually require modification of surgical technique for the second procedure. Dual-organ living donation is rare but can be safely performed independent of sequence.

## Graphical Abstract

**Figure:**
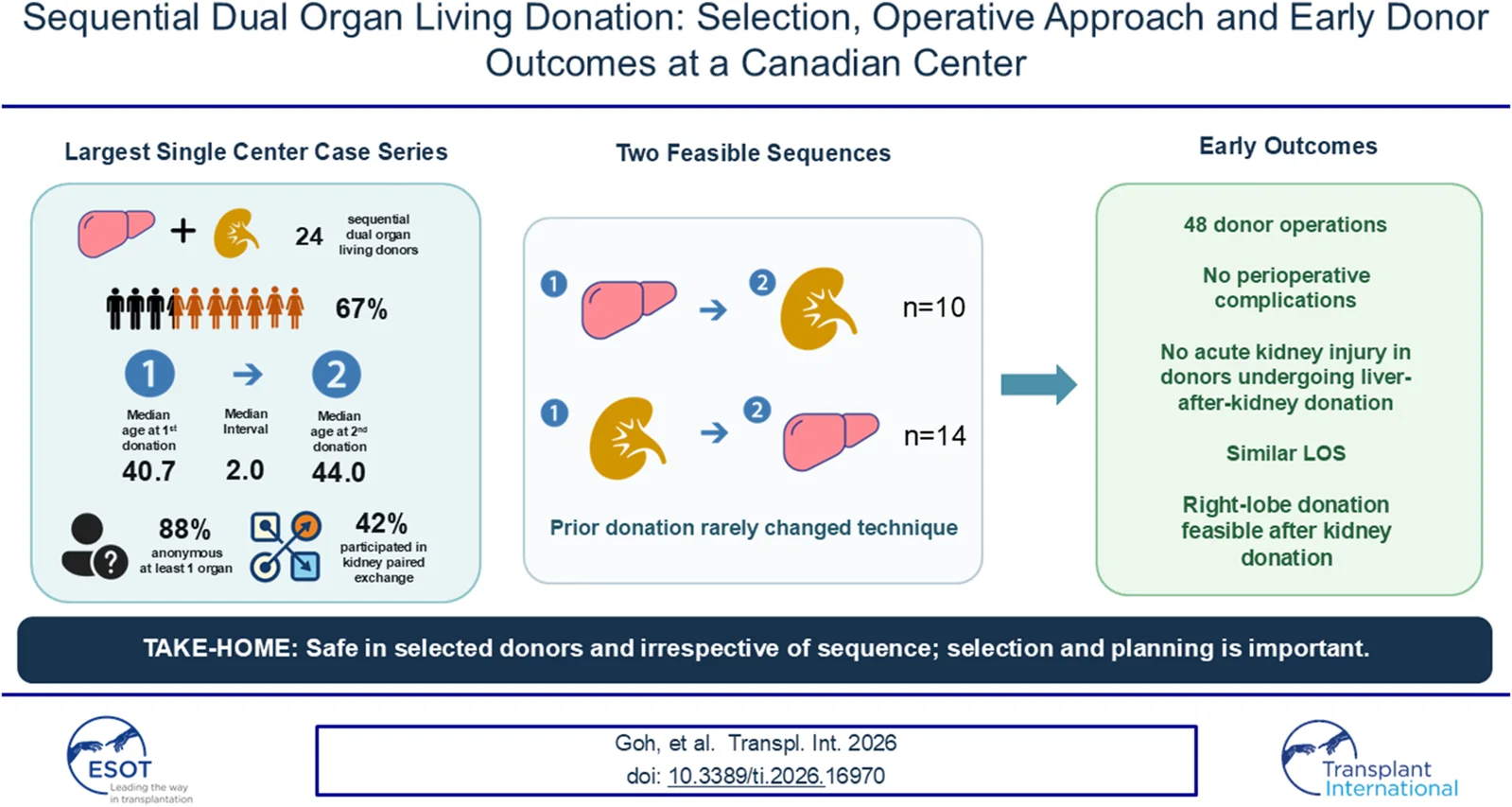

## Introduction

Living donation plays an important role in facilitating timely access to high-quality liver and kidney grafts for those in need of transplantation. However, unlike deceased donation, it requires a healthy individual to assume the operative risks for the benefit of another, making donor safety the fundamental constraint on the expansion of living donor transplantation. The ethical framework established at the Vancouver Forum [1] together with the Declaration of Istanbul on Organ Trafficking and Transplant Tourism anchor the principles of all living donation and set the standard against organ trafficking, tourism, and exploitation. Any extension of living donation must therefore remain consistent with these principles wherever it is practiced.

Within the living donation landscape, a small number of living donors extend this commitment to donate a kidney at one time and a partial liver graft at another or *vice versa*. This practice, termed sequential dual-organ living donation, is uncommon but important. It challenges assumptions derived from single-organ donation and requires renewed attention to ethical considerations of “double equipoise,” donor selection, timing between operations, and risk communication [2].

Emerging registry [3] and cohort data [4–9] report acceptable donor-specific outcomes. These reports have described both sequences (liver-first donation followed by kidney donation or *vice versa*) and outline perioperative outcomes that are acceptable in well-selected donors and within highly experienced teams. They also highlight the need for careful preoperative assessment, meticulous intraoperative technique, and structured early postoperative monitoring [10].

However, these reports have not included detailed information about surgical considerations and operative approach towards the second organ donation in prior donors. Furthermore, some authors have raised concerns about the safety of liver-after-kidney donation and have suggested that the amount of liver resected should be minimized in prior kidney donors to decrease the risk of perioperative renal dysfunction [10].

Herein, we present a donor-specific analysis and sequence comparison (liver-after-kidney and kidney-after-liver donation) of the largest single-center cohort of sequential dual-organ living donors reported to date from a high-volume North American living donation program. We describe (i) the demographic characteristics and donation patterns, directed versus non-directed, of these donors; (ii) the operative considerations posed by a second donor operation; and (iii) short-term perioperative donor outcomes.

## Methods

### Study design and setting

A retrospective review of all sequential dual-organ living donors at the Ajmera Transplant Centre, Toronto General Hospital, University Health Network (UHN), Toronto, Ontario, Canada was performed. The study period began at program inception and extended through December 2025. All living donors provided written informed consent for data collection under protocols approved by the UHN Research Ethics Board.

This study was designed as a donor-focused analysis. Sequential dual-organ living donors were defined as individuals who completed two separate living donor operations: one living donor hepatectomy (left lateral segment, left lobe, or right lobe) and one living donor nephrectomy (left or right sided, laparoscopic or open). Outcomes of interest (detailed below) included donor demographic characteristics, the nature and sequence of each donation, the interval between them, participation in kidney paired donation, operative approach, perioperative complications, length of stay, and perioperative renal biochemistry. Recipient characteristics and post-transplant recipient outcomes were not collected as the focus of this study was to evaluate the perioperative outcomes of sequential dual donors in relation to their sequence of donation.

### Protocol for selection – donor hepatectomy and donor nephrectomy

None of the donors in our cohort expressed a desire to donate two organs at the outset. The sequence of organ donation was therefore determined by individual donors without input from our team, and they were evaluated for either liver or kidney donation first based upon their initial presentation as willing liver or kidney donors, respectively. Evaluation for the subsequent living organ donation was conducted sequentially and independently of the first at the request of the donor. Evaluations for liver and kidney donation were conducted according to standard protocols, as previously described [11–13]. The medical, nursing, and psychosocial teams performing the evaluation of liver and kidney donors at our center are separate and distinct. The surgeons involved in living liver donation procedures and living kidney donation procedures are also distinct except for one surgeon who performs both.

Selection of which kidney to donate was driven by relative function. If function was symmetric, the anatomically less complex kidney was chosen. For liver donation, the type of hepatectomy was selected to maximize the donor’s functional liver remnant (FLR) and provide an adequate graft-to-recipient weight ratio (GRWR). If the left lobe (LL) or left lateral segment (LLS) anatomy was not favorable for donation, or if a suitable recipient for a LL/LLS graft was not available within the donor’s donation timeline, then right lobe donation was considered. Prior organ donation was not a factor in determining the laterality of liver or kidney donation.

Voluntary participation in Canada’s national kidney paired donation (KPD) program was offered to all anonymous nondirected kidney donors as well as directed living kidney donors who were found to be incompatible with their intended recipients.

### Operative procedures

All living donor hepatectomies were performed through an upper midline incision. Strict intravenous fluid restriction was implemented during all donor hepatectomy procedures to minimize central venous pressure, regardless of prior kidney donation status. Blood pressure targets were achieved with vasopressor administration until parenchymal transection was completed, after which resuscitation with intravenous crystalloids was initiated and vasopressors were rapidly weaned off. All living donor nephrectomies were performed laparoscopically, except in two in whom a planned open mini-incision was undertaken because of anticipated adhesions related to prior hepatectomies.

### Data collection

Variables included donor demographics and age at each donation, donor body mass index (BMI), comorbidities (presence of diabetes or hypertension), order of donation (liver-first vs. kidney-first), interval between donations, nature of each donation (directed vs. anonymous), participation in KPD, surgical approach and side/segment donated, length of stay, perioperative complications for each procedure, and perioperative renal biochemistry.

### Statistical analysis

Descriptive statistics were used for demographics and operative characteristics. Continuous variables were summarized using medians and interquartile ranges as appropriate. Between-group comparisons were assessed using the Mann-Whitney U test. Statistical significance was defined *a priori* as p < 0.05.

## Results

### Cohort characteristics

We identified 24 sequential dual-organ living donors during the study period, representing approximately 0.5% of all living donors in our center. Donors were a median of 40.7 years old at the time of their first donation (IQR 29.9–48.2 years) and 44.0 years old at the time of their second donation (IQR 32.5–49.8 years). The median interval between donations was 2.0 years (IQR 1.1–4.2 years). A majority of the donors were female (67%). Median donor BMI was 24.3 (IQR 22.8–27.6). None of the donors had hypertension or diabetes mellitus. Detailed cohort characteristics are summarized in Tables 1, 2.

**TABLE 1:** Characteristics of all dual living donors in this study.

Year of 1st donation	1st organ	Age at 1st donation (years)	Age at 2nd donation (years)	Interval (years)	Sex	BMI	Liver type	Approach Incision	Liver recipient	Liver LOS (days)	Kidney side	Approach Incision	Kidney Recipient	KPD	Kidney LOS (days)
2013	Kidney	40.0	49.8	9.8	F	22.3	RL	Open midline	Anonymous nondirected	6	L	Laparoscopic pfannenstiel*	Anonymous nondirected	N	3
2014	Liver	41.4	42.6	1.1	M	19.2	LLS	Open midline	Directed	8	L	Open Mini-incision	Directed	N	5
2014	Kidney	20.3	21.6	1.3	M	23.7	LLS	Open Midline	Anonymous nondirected	4	L	Laparoscopic pfannenstiel*	Anonymous nondirected	N	2
2015	Liver	19.8	25.1	5.3	F	28.3	LLS	Open Midline	Anonymous nondirected	5	L	Laparoscopic pfannenstiel	Anonymous nondirected	Y	3
2016	Kidney	30.1	40.0	9.9	F	27.8	RL	Open Midline	Anonymous nondirected	5	L	Laparoscopic pfannenstiel*	Directed	Y	3
2016	Liver	43.4	45.3	1.8	F	24.2	LL	Open Midline	Anonymous nondirected	6	L	Laparoscopic pfannenstiel	Anonymous nondirected	N	4
2016	Kidney	37.5	40.0	2.5	F	26.4	LLS	Open Midline	Directed	5	R	Laparoscopic pfannenstiel	Directed	N	4
2019	Liver	55.6	57.3	1.7	M	25.2	LLS	Open midline	Anonymous nondirected	4	L	Laparoscopic pfannenstiel	Anonymous nondirected	Y	3
2019	Kidney	37.4	42.8	5.4	M	35.3	LL	Open midline	Anonymous nondirected	5	L	Laparoscopic pfannenstiel*	Directed	N	3
2019	Liver	29.4	31.9	2.5	M	22.9	RL	Open midline	Anonymous nondirected	4	R	Open Mini-incision	Directed	N	2
2020	Liver	20.0	23.9	3.8	M	27.1	RL	Open midline	Anonymous nondirected	6	L	Laparoscopic pfannenstiel	Anonymous nondirected	N	4
2020	Kidney	26.3	30.4	4.1	M	24.3	RL	Open midline	Anonymous nondirected	4	L	Laparoscopic pfannenstiel	Anonymous nondirected	N	2
2020	Kidney	48.6	53.2	4.6	F	26.4	LL	Open midline	Anonymous nondirected	4	L	Laparoscopic pfannenstiel*	Anonymous nondirected	N	2
2020	Liver	53.5	54.4	0.9	F	21.5	LLS	Open midline	Anonymous nondirected	6	L	Laparoscopic pfannenstiel	Anonymous nondirected	Y	4
2021	Kidney	47.7	49.7	2.0	F	22.6	LLS	Open midline	Anonymous nondirected	5	L	Laparoscopic pfannenstiel	Directed	Y	4
2021	Liver	23.9	24.5	0.6	F	23.6	LLS	Open midline	Anonymous nondirected	4	L	Laparoscopic pfannenstiel	Anonymous nondirected	Y	2
2022	Liver	44.5	46.1	1.6	F	23.7	LLS	Open midline	Anonymous nondirected	4	L	Laparoscopic pfannenstiel	Directed	Y	2
2022	Kidney	48.8	49.9	1.1	F	27.4	LL	Open midline	Anonymous nondirected	4	L	Laparoscopic pfannenstiel*	Directed	N	2
2022	Kidney	53.1	55.4	2.3	F	31.6	RL	Open midline	Directed	7	L	Laparoscopic McBurney*	Directed	N	1
2022	Kidney	30.8	32.6	1.9	F	18.7	LL	Open midline	Anonymous nondirected	4	L	Laparoscopic pfannenstiel*	Directed	N	2
2022	Liver	43.1	45.2	2.1	F	24.3	RL	Open midline	Anonymous nondirected	7	L	Laparoscopic pfannenstiel	Directed	Y	5
2023	Kidney	32.0	33.0	1.0	F	31.6	LLS	Open midline	Anonymous nondirected	4	L	Laparoscopic McBurney*	Anonymous nondirected	Y	1
2023	Kidney	51.6	52.3	0.7	F	20.5	RL	Open midline	Anonymous nondirected	5	L	Laparoscopic McBurney*	Anonymous nondirected	Y	3
2024	Kidney	48.1	49.0	0.9	M	33.2	LL	Open midline	Anonymous nondirected	5	R	Laparoscopic pfannenstiel	Directed	N	3

*denotes donors who underwent kidney donation at another center prior to liver donation at our center. LLS: left lateral segment. LL: left lobe. RL: right lobe. KPD: kidney paired donation. LOS: length of stay. BMI: body mass index.

**TABLE 2:** Baseline characteristics of all sequential dual living organ donors.

Characteristics	Donors (n = 24)
Sex Male Female	8 (33%) 16 (67%)
Liver first, followed by kidney	10 (42%)
Kidney first, followed by liver	14 (58%)
Age at first donation	40.7 years (IQR 29.9–48.2)
Age at second donation	44.0 years (IQR 32.5–49.8)
Interval between donation	2.0 years (IQR 1.1–4.2)
Age at liver donation	42.9 years (IQR 32.1–49.8)
Liver donation type Right lobe Left lobe Left lateral segment	8 (33%) 6 (25%) 10 (42%)
Liver recipient type Anonymous nondirected Directed	21 (87%) 3 (13%)
Liver donation approach Open	24 (100%)
Age at kidney donation	41.3 years (IQR 30.6–48.2)
Kidney donation type Right Left	3 (12%) 21 (88%)
Kidney donation approach Open Laparoscopic	2 (8%) 22 (92%)
Kidney recipient type Anonymous nondirected Directed	12 (50%) 12 (50%)
Kidney paired donation	10 (42%)

### Donation sequence and nature of donation

Of the cohort, 42% (10/24) underwent liver donation first followed by kidney donation. The remainder of the cohort (14/24) donated a kidney first and subsequently underwent living donor hepatectomy. A high proportion of donors (88%, n = 21) performed at least one donation anonymously. Twelve living donors donated both organs in an anonymous nondirected fashion, nine donated one organ anonymously and directed the other, and three directed both donations. Twelve of 24 kidney donations were anonymous nondirected while 21 of 24 liver donations were anonymous nondirected. Ten out of 24 donors (42%) participated in KPD, thereby enabling additional transplants. Ten donors underwent living kidney donation at another transplant center in Canada prior to seeking liver donation at our center; nine of ten sought anonymous nondirected liver donation, while one was directed. All liver donation procedures were performed at our center. No liver-first donors subsequently donated a kidney at another transplant center in Canada.

### Operative characteristics

Liver donations included left lateral segment hepatectomy (n = 10), left lobe hepatectomy (n = 6), and right lobe hepatectomy (n = 8). All hepatectomies were performed through an upper midline incision. As discussed earlier, graft selection was individualized. In the kidney-first cohort (n = 14), five donated an RL graft, four donated an LLS graft, and five donated an LL graft. In the liver-first cohort (n = 10), three donated an RL graft, six donated an LLS graft, and one donated an LL graft.

Kidney donations were predominantly left-sided (88%). The laparoscopic approach was used in all except two donors. The first kidney-after-liver donor in our series underwent a planned open left nephrectomy due to anticipated adhesions from prior left lateral hepatectomy and lack of confidence about attempting a laparoscopic nephrectomy at an early stage in the evolution of our program. The only individual to donate a right kidney after right hepatectomy underwent a planned open right nephrectomy due to anticipated adhesions to the cut surface of the liver in the right upper quadrant that could complicate a laparoscopic approach.

### Perioperative outcomes and recovery

**FIGURE 1:**
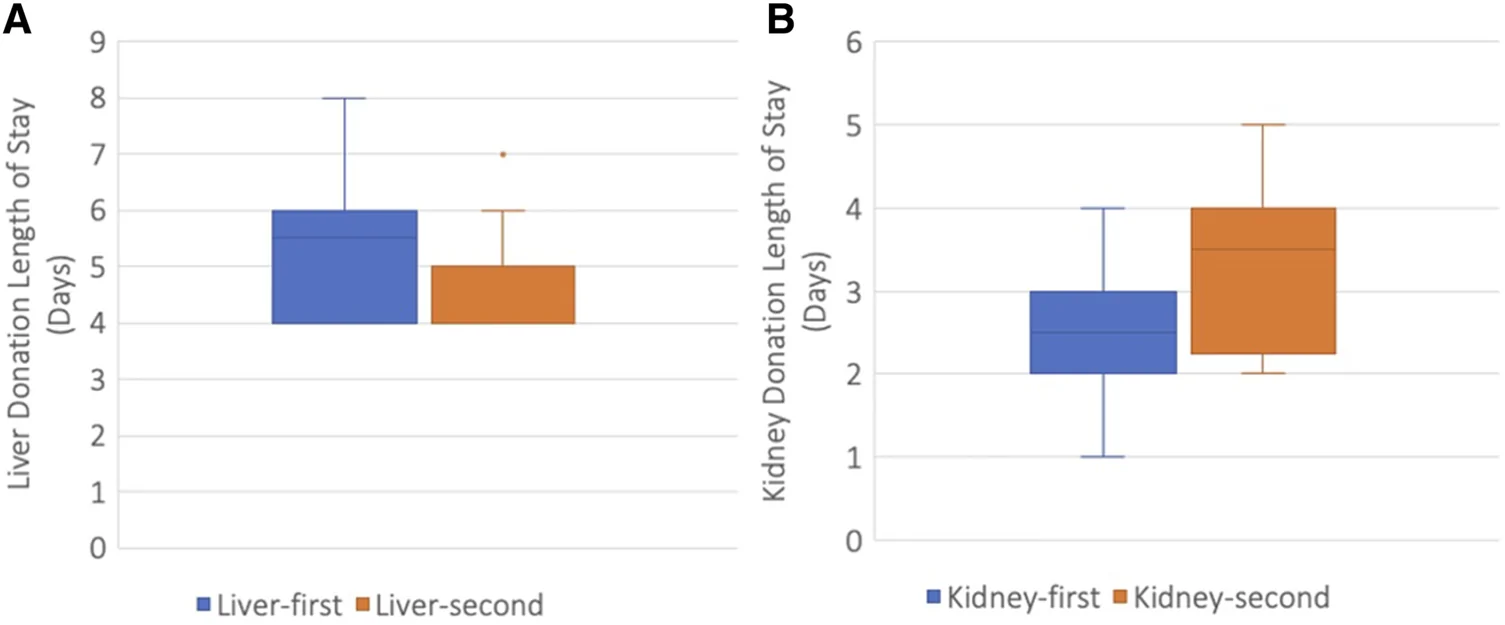
Length of stay for donors who underwent **(A)** liver-first (n = 10) compared to liver-second (n = 14) living donation. Mann-Whitney U p = 0.42 and **(B)** kidney-first (n = 14) compared to kidney-second (n = 10) living donation. Mann-Whitney U p = 0.07.

**FIGURE 2:**
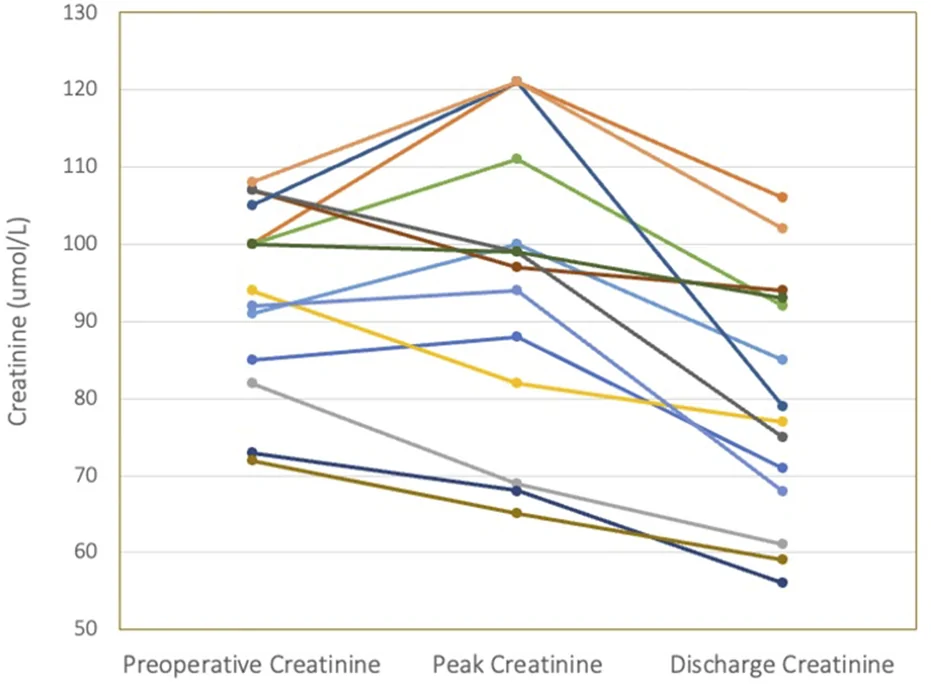
Creatinine levels of living donors who underwent living liver donation after living kidney donation (n = 14). Friedman test p= < 0.001.

None of the donors experienced a perioperative complication in either of their two procedures. The median LOS after living donation of a liver graft was 5.5 days (IQR 4.0–6.0) in the liver-first cohort and 5.0 days (IQR 4.0–5.0) in the liver-second cohort (Figure 1A). The LOS did not differ significantly (p = 0.42). The median length of stay (LOS) after living donation of a kidney graft was 2.5 days (IQR 2.0–3.0) in the kidney-first cohort and 3.5 days (IQR 2.3–4.0) in the kidney-second cohort (Figure 1B). This finding was not statistically significant (p = 0.07). None of the donors experienced acute kidney injury post donation (Figure 2). The type of donor hepatectomy performed did not impact renal function in prior kidney donors.

## Discussion

### Donor characteristics and perioperative outcomes

This is the largest single-center series of 24 sequential dual-organ living donors reported to date. Our granular series describes a donor population that is young, predominantly female, uniformly free of hypertension and diabetes, and characterized by a high prevalence of anonymous donation.

Twenty-one of 24 donors (88%) performed at least one donation anonymously and 12 donated both organs anonymously. This reflects the systematic development of anonymous living liver donation at our center [14–16]. As such, this led a substantial proportion of our cohort to seek liver donation here after donating a kidney elsewhere.

Across 48 donor operations there were no perioperative complications, length of stay did not differ significantly by donation sequence, and none of the liver-after-kidney donors developed acute kidney injury. Five liver-after-kidney donors underwent right lobe donation without perioperative renal impairment despite highly restrictive intraoperative fluid administration, consistent with earlier reports of preserved renal function in this sequence [5, 10]. This does not support the suggestion that the volume of liver resected should be constrained in prior kidney donors [10]. However, the small sample size precludes an accurate estimation of risk, and these findings should be cautiously extrapolated to the broader population of prior living kidney donors.

These uniformly favorable outcomes should be interpreted in the context of an exceptionally healthy and highly selected donor cohort. All donors were fit and free of comorbidities. In donors returning for a second donation, more stringent thresholds for fitness and risk should apply.

### Implications for donor selection, sequencing, and operative planning

Prior donor surgery rarely altered the operative approach to the second donation (kidney-first, n = 14; liver-first, n = 10). Laparoscopic nephrectomy was feasible in all prior liver donors except two, early in our experience, in whom a planned open mini-incision was undertaken for anticipated adhesions from ipsilateral prior hepatectomy with an unsuitable contralateral kidney.

Conversely, prior kidney donation did not influence the type of hepatectomy performed. Graft selection followed the usual principles of maximizing the donor’s FLR while providing a graft of adequate graft-to-recipient weight ratio. Prior donation should therefore inform operative planning and consent rather than constrain graft type or laterality.

No donor identified dual donation as an objective at first presentation. Each returned of their own accord. As such, the sequence observed reflects the order of presentation rather than the design of our program. For a candidate presenting with dual donation as an objective, our data do not support a prescriptive pathway.

Each sequence carries theoretical trade-offs: kidney-first places the lower-risk operation first, whereas liver-first avoids a complex hepatectomy in a donor with a solitary kidney. Sequence selection is best grounded in donor wishes, anatomy and physiologic reserve, recipient urgency, and center experience, with equipoise maintained across both options.

A median interval between donations of 2.0 years was observed in our cohort. Our current data at this point are insufficient to define a minimum interval policy between donations. However, a minimum interval of 6 months was considered appropriate to allow adequate physiological recovery and reassessment of psychosocial stability before a second donation.

Ten of 24 donors (42%) participated in kidney paired donation, so the yield from one individual’s decision may extend beyond two transplants, and integration of living liver donors into liver-paired exchange could extend it further [17]. No consensus guidance exists for donors who return, and we would support standards addressing selection, minimum interval, consent for cumulative risk, and a registry able to audit the sequential donation journey.

### Limitations

Limitations include the retrospective design, modest sample size, and the lack of long-term quality of life and functional outcomes. Follow-up was confined to the perioperative period, so long-term renal and hepatic function and the cumulative long-term risk of two donor operations remain unaddressed. We did not examine psychosocial drivers, donor identity, or health-related quality of life, all of which shape attitudes to donation [18], and our observations regarding anonymous donation are therefore descriptive rather than explanatory. This study was designed as a donor-specific analysis; therefore, recipient characteristics and post-transplant outcomes were not examined, nor was a comparator cohort of single-organ donors included. All of the above would be valuable in future multicenter studies to better contextualize the outcomes of sequential dual-organ donation.

## Conclusion

Sequential dual-organ living donation is rare but was completed safely in this cohort irrespective of sequence, and prior donation seldom required modification of operative technique. For programs contemplating sequential donation, our data support disciplined selection, thoughtful planning, and vigilant perioperative care. Early donor outcomes were reassuring, including preserved renal function after right lobe donation in prior kidney donors. The field now needs consensus guidance specific to these donors and registry infrastructure capable of following them beyond the perioperative period. The cumulative impact of these donors on transplant activity, including their role in paired donation chains, justifies the investment.

## Data Availability

The original contributions presented in the study are included in the article/supplementary material, further inquiries can be directed to the corresponding author.
